# Potential distribution of *Aedes vittatus* as an invasive species in North America

**DOI:** 10.1371/journal.pone.0335534

**Published:** 2025-12-05

**Authors:** Eric Ng'eno, Claudia Nuñez-Penichet, Zenia P. Ruiz-Utrilla, Paula Isla Martins, Victoria Argudo, Weverton C. F. Trindade, Narayani Barve, Abdelghafar Alkishe, A. Townsend Peterson

**Affiliations:** 1 Biodiversity Institute, University of Kansas, Lawrence, Kansas, United States of America; 2 Fish and Wildlife Conservation, Virginia Tech, Blacksburg, Virginia, United States of America; 3 Laboratório Ecologia da Intervenção, Instituto de Biociências, Universidade Federal de Mato Grosso do Sul, Campo Grande, Mato Grosso do Sul, Brazil; 4 Laboratory of Plant Ecology, Federal University of Paraná, Curitiba, Paraná, Brazil; 5 Department of Ecology and Evolutionary Biology, University of Tennessee, Knoxville, Tennessee, United States of America; World Health Organization, Regional Office for South-East Asia, INDIA

## Abstract

The recent detection of populations of the mosquito *Aedes vittatus* in the Dominican Republic and Cuba has raised concerns over its potential for broader invasion in the Western Hemisphere, and particularly on the North American mainland. This species has been associated with the maintenance and transfer of yellow fever, Zika, chikungunya, and dengue viruses on its native distributional area. A previous study identified parts of North America with environments apparently suitable for the species, but different modeling approaches can give different results. Using models calibrated across the region historically accessible to the species, we re-examined the distributional potential of this species. Our models identified areas with year-round environmental suitability for this species along the southern coasts of the US, Mexico, Caribbean islands, and Central America. These environmentally suitable areas coincide with those for other *Aedes* species and overlap with urban settings, potentially placing large human populations at increased risk in case of successful establishment and invasion by this species. Targeting entomological surveillance in these and nearby areas is important for early detection to avoid establishment of populations of this species.

## Introduction

Mosquito-borne arboviruses account for a substantial burden of disease globally [1–3]. Although inadequate surveillance systems in many settings likely hinder accurate estimation, the disease burden associated with mosquito-borne diseases is considerable. In 2023 alone, ~ 6.5 million cases and 7300 deaths resulted from dengue fever [4], and 460,000 cases and 360 deaths resulted from chikungunya virus (largely in the Americas) [5,6]; 2400 and 700 West Nile virus cases were reported from the US and Europe, respectively [6,7], and 27 laboratory-confirmed yellow fever virus cases were reported from Africa [8].

The viruses mentioned above are transmitted in largest part by mosquitoes of the genus *Aedes*, several of which have undergone major range shifts and extensions, owing to human-assisted dispersal, climate change, and urbanization [9]. *Aedes aegypti*, which transmits dengue, chikungunya, yellow fever, and Zika viruses, and *Ae. albopictus*, which transmits dengue, chikungunya, Zika, and West Nile viruses, are considered principal vectors due to their vectorial competence and anthropophilic traits [10–12]. Consequently, several studies have assessed their susceptibility to different viruses [13], and potential distributions globally [9,14,15].

Recent studies have shown that virus transmission in human populations is likely also modulated by the presence of other *Aedes* species of relatively lower vectorial competence, but that may bridge between sylvatic and human transmission cycles [10,16–18]. These species can maintain virus populations between outbreaks in human population through sylvatic cycling or via vertical transmission [19–21]. Occasionally, these species may even trigger outbreaks, such as when viral mutations improve vectorial competence [22–24], and by exhibiting context-dependent behavioral shifts that may increase anthropophilic preferences [25–27]. Knowledge of the distributions of these other species, which potentially can interact synergistically with primary vectors (though negative vector interactions have also been described [28]) is therefore important.

*Aedes vittatus,* a species native to tropical regions of Africa and Asia, is an example of a bridge vector and has been has been documented as a significant and competent vector of yellow fever, Zika, chikungunya, and dengue viruses [16]. It has a short life cycle, survives under high temperatures (> 40°C) and dry conditions, shows adaptability to diverse breeding habitats, and feeds on both humans and animals. These traits are important for its population growth, sustenance, and geographic expansion and may make it important in public health considerations [16,29–31].

Populations of *Ae. vittatus* were recently documented in Jarabacoa, Dominican Republic, and Guantanamo Bay, Cuba, suggesting successful introduction and establishment in these areas [32–34]. Establishment of this species in the Americas is raising concerns over its broader spread potential in the region owing to international trade, human movements, warming conditions, urbanization, and other socioeconomic factors that have been associated with distribution of other *Aedes* species [9].

A recent study exploring the global distribution of *Ae. vittatus* identified regions of Central America, the Caribbean, and southern US as presenting environmental conditions suitable for *Ae. vittatus* populations [35]. Here, we re-examine the species’ potential distribution to identify areas for targeting entomological surveillance and interventions in the US and elsewhere. Our approach differs from that of the previous study in that we restricted model calibration explicitly to areas to which the species likely has had access [36,37]; we also balanced occurrence data to a more even density across the native distributional areas for model calibration. We implemented detailed post-modeling analyses to characterize areas of extrapolation where model predictions may be less dependable [38].

## Methods

### Occurrence data

We compiled all georeferenced occurrence data available for *Ae. vittatus* as of 11 October 2023. The data were sourced from the Global Biodiversity Information Facility (https://www.gbif.org/, doi.org/10.15468/dl.qwu8nd), VectorMap (2024, https://wrbu.si.edu/), and Barcode of Life Data Systems (BOLDSYSTEMS, https://v3.boldsystems.org) databases, and from peer-reviewed publications [32,33,39–50]. Data with date information span the period 1907–2024. We transformed coordinates from diverse original formats into decimal degrees. We cleaned the data by removing duplicates, occurrences with “0,0” coordinates, and records with low precision (i.e., fewer than 2 decimal places), following Cobos *et al* [51]. We compared coordinates of occurrence records with those of country centroids, and excluded records that had been assigned centroid coordinates or that were within a 5 km radius of a country centroid. We also excluded records falling outside the limits of the country described textually in the data record. A few (four) records falling outside of the extent of the environmental layers (particularly at the edge of terrestrial areas) but within 5 km of land were moved to the nearest pixel with data.

To ameliorate potential effects of spatial autocorrelation and sampling biases related to potential differences in survey intensity, data sharing, and data availability among countries, we conducted two-levels of spatial thinning. First, we performed a spatial thinning, in which we retained only single occurrence records within a 5 km radius [52] using the *spThin* R package version 0.2.0 [53]. Second, we performed a country density-based thinning, in which we determined the densities of occurrence points per unit area (square miles) for every country in the native range for the species [54]. Country point densities were calculated by dividing total numbers of spatially thinned occurrences in a country by the country’s area. We then determined the median density across all of the countries, which we used as a reference density in succeeding steps.

We multiplied the median density across countries by each country’s total area in square miles to generate an expected number of occurrences. If the number of expected occurrences was higher than the number of records available, we included all points available from that country. If the expected number of occurrences was lower than the number available from that country, however, we used a reduced number of records in the analysis (countries that met this criterion included Benin, Central African Republic, Comoros, Dominican Republic, Djibouti, Guinea-Bissau, Kenya, Liberia, Madagascar, Nigeria, Spain, Sri Lanka, Thailand, and Uganda). The reduced number of occurrences was randomly sampled from the pool of reported occurrences in each country. To account for potential biases that could arise from selection of a reduced number of occurrences to represent the species’ occurrence in a country, we generated 10 replicate sets of subsampled datasets.

### Environmental variables

We obtained environmental (climatic) variables from the WorldClim database (https://www.worldclim.org) at 2.5’ spatial resolution (~4.6 km at the Equator). We removed the variables mean temperature of wettest quarter (Bio 8), mean temperature of driest quarter (Bio 9), precipitation of warmest quarter (Bio 18), and precipitation of coldest quarter (Bio 19), as they are known to contain spatial artifacts as a result of combining temperature and precipitation information [55,56]. Based on the 15 remaining environmental variables, we generated principal components (PCs) to reduce dimensionality and multicollinearity among environmental dimensions [57].

### Area for model calibration

We used a simulation approach to determine the calibration area (i.e., the area that has been accessible to a species over relevant time periods) [36,37]. In brief, for these simulations, a subset of occurrence records is selected randomly for use as starting populations in the simulation. Dispersers move from these populations (cells) into other cells based on a probability density function (dispersal kernel) that broadly defines cells comprising the catchment area of dispersing individuals from occupied cells. Dispersers can also fail to leave a cell. Coordinates of cells to be accessed are defined based on dispersal distances drawn from the dispersal kernel and dispersal angle is derived from a random distribution. Cells invaded are colonized if they present suitable environmental conditions and *vice versa*, where suitability is defined using past or current climatic conditions. The colonized cells, including the initial cells, then become dispersing cells from which new cells within their catchment are tested for suitability. Cells with larger populations (more suitable) disperse to more cells than those with smaller populations. The dispersal process is iterated based on the number of dispersal events defined *a priori*.

The full simulation is replicated multiple times using different randomly selected starting populations and different parameter settings, and the range of areas accessible by a species is determined as the most accessed areas, whether they are colonized or not. The simulation was implemented in the *grinnell* R package, version 0.0.22, [36]. We limited the simulation to the Eastern Hemisphere (Europe, Africa, and Asia), a large part of which is considered as part of the native distribution of *Ae. vittatus*; other parts, though perhaps not part of the species’ native distribution, have already been colonized successfully, and thereby can be used as useful data inputs in ecological niche models [16,58] –the key point is that the species is in distributional equilibrium to a greater degree in Eurasia and Africa than in the Americas, where it is newly arrived. To increase the probability of sampling points into the starting population and ensure variability in the number of species dispersing from each cell during the simulation, we used 90% of the occurrence points as the starting population, set maximum dispersers at 5, and number of iterations at 10. For lack of empirical data on dispersal for this species with which to constrain our parameter values, we explored a range of plausible dispersal events and distances. We explored dispersal event numbers including 30, 60, 120, 180, and 240 events, using normal dispersal kernels with standard deviations ranging at integer values between 1 and 8. Suitability was based on current climatic conditions, in view of the recent range dynamics of this species. We implemented the simulation for each of the 10 data subsets, and assessed each simulation for convergence in the simulated dispersal range. We merged recently invaded countries (i.e., Cuba, Dominican Republic) manually into the simulated accessible area, and used the resulting area as the final extent for model calibration.

### Model calibration and selection

Model calibration and selection were implemented using maximum entropy modeling (Maxent) approaches [59,60] via the *kuenm* R package version 1.1.10 [61]. In brief, for each subset, we used a random 70% of available data for model training, and reserved the remaining 30% for model testing. We generated multiple candidate models considering all possible combinations of two or more of the PCs, diverse regularization multiplier values (0.10, 0.25, 0.50, 0.75, 1, 2, 3, 4, 5), and diverse combinations of feature classes [all combinations of linear (l), quadratic (q), and product (p)]. We selected candidate models based on (1) statistical significance (calculated from partial receiver operating characteristic curves [62], with 500 iterations and 50% of data for bootstrapping); (2) omission rate (5% criterion) [63]; and (3) Akaike Information Criterion corrected for small sample sizes (AICc; only models with ΔAICc ≤ 2 were included in the final analysis [64]).

We created final models for each of the 10 occurrence datasets, using parameters of the candidate models that passed all evaluation filters. The models were bootstrapped 10 times using a jackknife process with cloglog values as the output. We transferred the final models across space (i.e., first to the calibration area to determine how well we recover known areas of occurrence, and then more broadly) under free extrapolation, extrapolation with clamping, and no extrapolation [38]. As a fourth criterion for model inclusion, we inspected the response curves with respect to each environmental dimension; we excluded from further analysis any models with bimodal response curves, as fundamental ecological niches are not likely to have this type of response [65]. If we had more than one final model for a dataset, we created a consensus model by calculating the median of all final models.

To determine areas potentially suitable and unsuitable for the species, we binarized the final models (0: unsuitable, 1: suitable). To this end, for single models, we extracted suitability values for each of the occurrence points used in model calibration, and considered the values in the lower 5% as unsuitable. In cases in which we created a consensus model, we used cloglog values from the 10 replicate models for all the models used to create the consensus and calculated the median. Those medians were sorted, and the lower 5% was classified as unsuitable.

### Occurrence points in environmental space

We checked the 10 occurrence datasets to identify potential variation in environments represented across different subsets, as these variations could lead to important differences in the models. To this end, we extracted values of the first two principal components (PCs) at each pixel containing the occurrence points, and across the full area that was accessible to the species (calibration area). These PCs were used as multivariate representation of the environmental conditions in these areas. We then visualized the distribution of environments where occurrence was detected *versus* those of the calibration area.

### Model uncertainty

We evaluated one important dimension of model uncertainty by assessing the risk of strict extrapolation when transferring onto non-analogous environmental conditions, such as across our broader area of interest in which we assessed the potential for further colonization and invasion by populations of *Ae. vittatus*. To this end, we employed the mobility-oriented parity (MOP) metric [38], which measures similarity between the closest 5% of environmental conditions from background points within the calibration area and each pixel within the transfer area. We conducted MOP assessments on each dataset, restricting analysis to the variables identified in the respective final models. Transfer areas with zero similarity values (i.e., those showing high MOP distances) to the calibration areas are identified as non-analogous, indicating higher uncertainty. In these regions, suitability predictions depend entirely on model extrapolation, requiring careful interpretation of species suitability. MOP analyses were conducted using the *mop* R package, version 0.1.2 [66]. All data cleaning steps and analyses were done in R version 4.2.2 [67].

## Results

**Fig 1:**
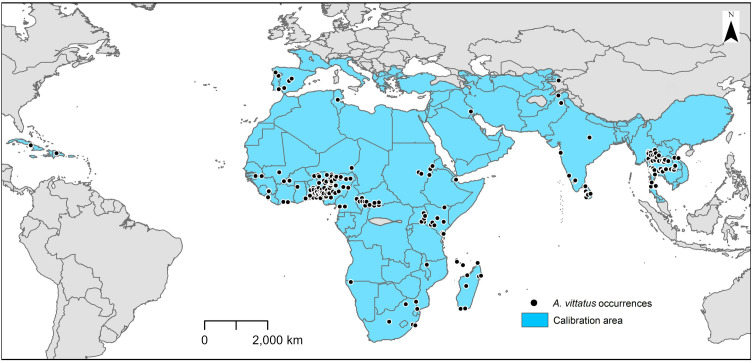
Assembled occurrence records of *Aedes vittatus.* Map showing all abstracted occurrence points (black dots) available to us for *Aedes vittatus*, and the calibration area (blue area) estimated for the species via simulations. Administrative boundaries were obtained from the database of global administrative areas (GADM), version 4.1, available at www.gadm.org.

In total, we extracted 320 occurrence records, of which 241 (75.3%) were included in analyses after the data cleaning and filtering steps. Records were distributed across tropical regions, including the Dominican Republic, Cuba, sub-Saharan Africa, India, and Thailand; the species was also found in subtropical regions, including Iraq, Pakistan, South Africa, and the western Mediterranean region (Italy, France, Spain, and Portugal); the species is also known from temperate Kyrgyzstan (Fig 1).

We retained the first six PCs for further analysis and exploration, as they explained 99% of the total climate variance across the calibration area (S1 Fig). PC1 explained the most variance (48%) followed by PC2 and PC3 with 30% and 10%, respectively. Temperature annual range (Bio 7), minimum temperature of the coldest months (Bio 6), temperature seasonality (Bio 4) and mean temperature of the coldest quarter (Bio 11), were the most influential variables, in order of decreasing importance (S1 Table).

Simulation models for estimation of accessible areas for *Ae. vittatus* converged at kernel spread of 8 and 240 dispersal events for all data subsets. Accessible area estimates included a region encompassing all occurrence data available except for the occurrences reported from Kyrgyzstan. The predicted accessible area included the whole of Africa; Madagascar; the Mediterranean region; and western, central and eastern Asia (Fig 1).

For model calibration, we evaluated a total of 5130 candidate models for each data subset, which resulted in a total of 108 selected final models. For nine of the data subsets, response curves with respect to the environmental axes were unimodal (i.e., either quadratic with downward concavity, or linear). However, for data subset 3, all four “best” models that resulted from assessment of candidate models had bimodal responses for PC1; as a result, data subset 3 and its four models were excluded from further analyses (S2 Fig), leaving 104 final models as a basis for the consensus model.

**Fig 2:**
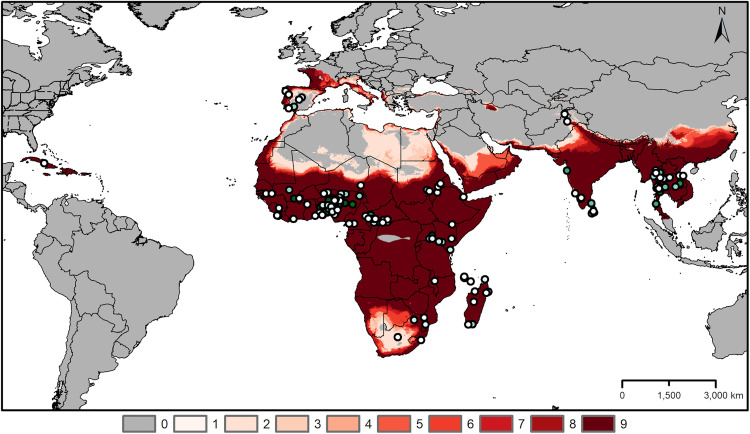
Areas predicted as having suitable environment in the region of model calibration. Areas predicted suitable for *Aedes vittatus* across the calibration area used in this study. Varying color intensity of the map reflects the consensus among different data subsets (darker shades indicating areas predicted as suitable by a greater number of subsets). Points show occurrences used in model calibration. Color indicates points included in more than one data subset. Predictions were generated with free extrapolation. Administrative boundaries were obtained from the database of global administrative areas (GADM), version 4.1, available at www.gadm.org.

The final consensus model identified areas with suitable environments in the Mediterranean regions of Europe and North Africa; sub-Saharan Africa; Middle East; Indian Subcontinent and Southeast Asia in the native-range predictions, as well as in Cuba and Dominican Republic (Fig 2).

**Fig 3:**
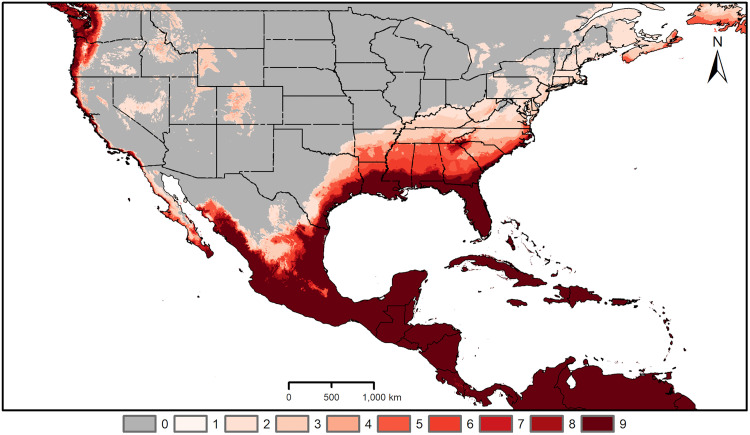
Areas predicted as having suitable environment in regions of model transfer. Areas predicted suitable for *Aedes vittatus* in the Caribbean, Central America, and North America. Varying color intensity of the map reflects the consensus among different data subsets (darker shades indicating areas predicted as suitable by a greater number of subsets). Predictions were generated with free extrapolation, but are closely similar to those generated with other assumptions about extrapolation (Fig 4). Administrative boundaries were obtained from the database of global administrative areas (GADM), version 4.1, available at www.gadm.org.

**Fig 4:**
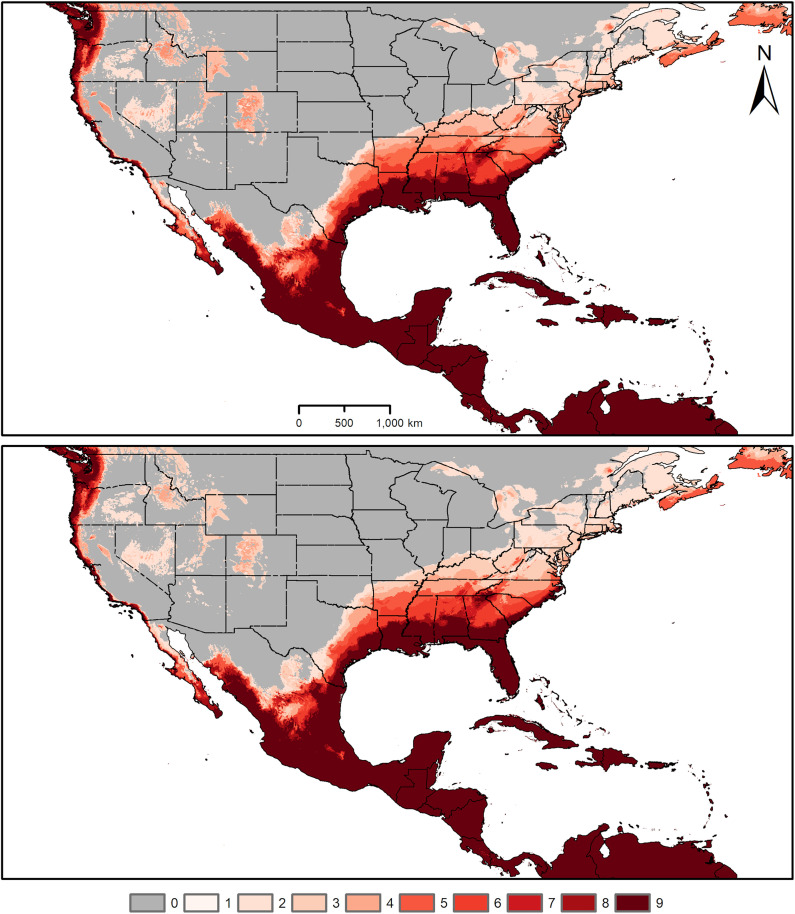
Extrapolation types used in model transfer. Areas predicted suitable for *Aedes vittatus* under assumptions of no extrapolation (top panel) and extrapolation with clamping (bottom panel). Varying color intensity of the map reflects the consensus among different data subsets (darker shades indicating areas predicted as suitable by a greater number of subsets). Administrative boundaries were obtained from the database of global administrative areas (GADM), version 4.1, available at www.gadm.org.

Across the Americas, where the species has potentially begun an invasion process, our models identified areas of high environmental suitability across the Caribbean, and in Central America, including Panama, Costa Rica, Nicaragua, Honduras, and Guatemala (Fig 3). Areas of high environmental suitability were also identified across large parts of Mexico. In the US, high environmental suitability was predicted along the Pacific, Gulf and southern Atlantic coasts. Predictions of environmental suitability did not vary markedly with different options regarding model extrapolation (Fig 4).

Exploration of the distribution of the occurrence data subsets in environmental space revealed variation in the environmental conditions represented among the different data subsets (S3 Fig). We could not detect patterns in that variation, and data subset 3 (which presented bimodal response curves) was not noticeably distinct.

When we examined extrapolation risk, which can emerge owing to differences between environments used for model calibration and those manifested across model transfer regions, we observed notable environmental dissimilarities in the area of transfer (S4 Fig). Specifically, extrapolation occurred towards higher values of PC5 in Mexico and Arizona (S5 Fig), and lower values of PC3 in Louisiana (S6 Fig).

## Discussion

In this analysis, we examined the environmental distribution of known occurrences of *Ae. vittatus* and used modeled summaries of that distribution to estimate its potential distribution in the Americas. Within the calibration area, our models included all countries where species has been identified [68], as well as countries in North Africa, Europe and West Asia where it has not yet been reported. Consistent with the results of a previous study [35], we observed year-round environmental suitability in coastal areas along the Gulf of Mexico in the US, and in neighboring regions of eastern, western, and southern Mexico, most of the Caribbean islands, and Central America. However, our predictions indicated a broader potential distribution in the US, Mexico, and Central America, which aligns with the known potential ranges of other species within the genus *Aedes*.

Several species of *Aedes* have successfully invaded the Americas over the years. *Ae. aegypti* was first introduced to the Americas around the 17th century, and has been linked to outbreaks of yellow fever and dengue [69,70]; it now has a broad invaded range across southern and southeastern US, and southward across the Neotropics [14], with sporadic detections farther southwest and northeast also reported [71]. *Ae. albopictus* is thought to have first been introduced into the Americas around 1985 [72], and carries arboviruses similar to those vectored by *Ae. aegypti*; it has a broader temperature tolerance and a consequently broader geographic range including the Pacific Coast and most of the eastern US [14]. Other non-native *Aedes* species in the Americas include *Ae. vexans* [introduced around 1930] and *Ae. japonicus* [introduced around 2000]; these species have tested positive for La Crosse virus, Zika virus, and West Nile virus, [73–75].

The diversity of mosquito species in an area can impact transmission of mosquito-borne pathogens [76]. That is, many mosquito-borne pathogens can be transmitted by multiple species, individual mosquito species can transmit different pathogens, and diverse mosquito communities can broaden vector-host-vector interactions so as to enhance pathogen persistence and transmission in an area [77]. The areas identified as suitable for *Ae. vittatus* coincided in large part with potential distributional areas for other *Aedes* species (particularly *Ae aegypti* and *Ae. albopictus*), and have large human presence and a considerable diversity of animal species [78], all of which could augment risk of pathogen maintenance and transmission; risk owing to the latter factors also depends on whether species present are efficient reservoirs for the viruses transmitted by these vectors [79,80].

*Ae. vittatus* is a mosquito species generally considered to inhabit forest and savanna [16]. It plays an important role in maintenance of sylvatic arbovirus cycles that occur commonly in Africa and Asia, but rarely in the Americas [19,81,82], in spite of some viruses demonstrating potential for sylvatic maintenance in the Americas [83,84]. A lack of a suitable forest-dwelling mosquito species could be one of limiting factors for viral outbreaks in the Americas; successful invasion and establishment of populations of *Ae. vittatus* may bridge this gap. However, the species’ effectiveness as a vector is also influenced by other factors, including viral adaptations to the mosquito and environmental conditions [85–87]. It therefore remains difficult to predict how an invasion by this species could alter the existing risk of arbovirus transmission to humans in novel distributional areas.

Our maps show suitable environments for the species extending the Gulf of Mexico into the southern Atlantic states. Assuming invasion from the neighboring areas where the population have recently established, two primary natural dispersal routes emerge: one along the Gulf of Mexico, and the other across the Atlantic from Cuba, the latter being a more parsimonious path even when accounting for potential human facilitation. Given the species’ versatility as regards breeding habitats [16], areas identified with year-round suitable environments, both in urban and rural settings, could be prioritized for entomological surveillance, particularly those areas with increased interactions with countries where the species has invaded.

This study nonetheless has several limitations. The results that we present are based on year-round environmental suitability estimates, yet environmental conditions vary over time, such that time-specific models may provide better characterizations of the potential distribution of the species, which has a remarkably short life cycle [88]. We also did not consider finer-resolution, habitat- or substrate-related factors that can create microclimates where mosquito populations can thrive or at least persist even under seemingly unsuitable climatic conditions [16]. Models incorporating such additional dimensions can provide fine-scale spatial details that may modify the broader patterns shared herein. However, since our goal is to define regions for heightened entomological surveillance for this potential invasive mosquito species, such broad patterns may be most appropriate. We did not account for biotic interactions: as shown previously [89,90], such interactions generally influence local-scale dynamics, but their effects diminish at broader spatial scales that are more relevant to this study. Further, biotic interactions can be population- and environment-specific, making their geography challenging to anticipate and incorporate in models such as those that have developed [91]. Incorporation of human movement and land use patterns into analyses could further refine potential distributional extents as well as potential routes of introduction.

## Conclusion

Modeling areas with year-round suitable environments for *Ae. vittatus* invasion identified coastal areas of west, southern and southeastern US, as well as broader areas to the south across the Neotropical region. These areas overlap broadly with those of the now-established populations of *Ae. aegypti* and *Ae. albopictus*; invasion by a new potentially complementary species could increase the burden of several mosquito-borne pathogens, particularly given the considerable human population residing in those areas. Although not a hyper-competent vector species, *Ae. vittatus* is anthropophilic, and has been shown to transmit yellow fever, Zika, chikungunya and dengue viruses on its native range. Targeting entomological surveillance in areas identified as environmentally suitable, as well as in neighboring areas that may present seasonal environmental suitability, could facilitate early detection and management of potential invasion by the species.

## Supporting information

S1 Table Summary of the loading of the principal components. (DOCX)

S1 Fig Summary of variance explained by the principal components. (PDF)

S2 Fig Response curve with respect to principal component1 (PC1) for model generated with data subset 3 and its projection over space in North America. (TIFF)

S3 Fig Distribution of *Aedes vittatus* occurrence records in environmental space. Each plot is one of the randomly sampled data subsets. The plots represent the first principal components (PC) of environments assessed. Each of the dots (gray and black) represents an existing combination of principle components, that is, an existing, unique, environment in the calibration area. The black dots represent the environments of location of reported occurrence of *Aedes vittatus*.
(TIF)

S4 Fig Overall extrapolation risk of model transfer onto areas with non-analogous environmental conditions. Figure shows estimates of Mobility Oriented-Parity distance which measures the dissimilarity between environments in area of model calibration and transfer. A distance of 0 indicates similar environments while distance of >0 indicate degree of dissimilarity between environments used in model calibration and those in areas of transfer. Basemap data sourced from global administrative areas (GADM), version 4.1, available at www.gadm.org.
(TIF)

S5 Fig Extrapolation risk of model transfer onto areas with non-analogous environmental conditions by specific variables. Figure displays dissimilarity estimates of specific environments towards the higher value. Dissimilarity estimates are based on Mobility Oriented-Parity distance. Basemap data sourced from global administrative areas (GADM), version 4.1, available at www.gadm.org.
(TIF)

S6 Fig Extrapolation risk of model transfer onto areas with non-analogous environmental conditions by specific variables. The figure shows dissimilary estimates of specific environments towards the lower value. Dissimilarity estimates are based on Mobility Oriented-Parity distance. Basemap data sourced from global administrative areas (GADM), version 4.1, available at www.gadm.org.
(TIF)
