# Supplementary material for: Potential distribution of Aedes vittatus as an invasive species in North America
Source: PLoS One. 2025 Dec 5;20(12):e0335534. doi: 10.1371/journal.pone.0335534 (PMC12680183; doi:10.1371/journal.pone.0335534)
Supplement: S1 Table — (DOCX) [file pone.0335534.s001.docx]

**S1 Table: Summary of the loading of the principal components**

| Environments | PC1 | PC2 | PC3 | PC4 | PC5 | PC6 |
| --- | --- | --- | --- | --- | --- | --- |
| Bio1 | 0.2863 | -0.3034 | -0.1346 | -0.0522 | -0.0542 | 0.0287 |
| Bio2 | -0.1598 | -0.2936 | 0.0012 | 0.1058 | 0.7754 | -0.3497 |
| Bio3 | 0.3166 | -0.0447 | 0.0904 | 0.3556 | 0.2974 | -0.0657 |
| Bio4 | -0.3352 | 0.0544 | -0.1455 | -0.3521 | -0.0199 | 0.0345 |
| Bio5 | 0.0939 | -0.4107 | -0.3046 | -0.3143 | 0.0283 | -0.0811 |
| Bio6 | 0.3385 | -0.1839 | -0.0655 | 0.1137 | -0.1354 | 0.0207 |
| Bio7 | -0.3418 | -0.0285 | -0.1044 | -0.3207 | 0.1759 | -0.0726 |
| Bio10 | 0.1493 | -0.3758 | -0.3029 | -0.3308 | -0.1036 | 0.0497 |
| Bio11 | 0.3274 | -0.2264 | -0.0382 | 0.1065 | -0.0362 | -0.0031 |
| Bio12 | 0.2925 | 0.2659 | 0.0423 | -0.2427 | 0.1154 | -0.1401 |
| Bio13 | 0.2785 | 0.1588 | 0.3082 | -0.3969 | 0.0548 | -0.2206 |
| Bio14 | 0.1770 | 0.3280 | -0.3937 | -0.0431 | 0.2972 | 0.3354 |
| Bio15 | -0.0064 | -0.2783 | 0.5251 | -0.1986 | 0.2465 | 0.7303 |
| Bio16 | 0.2835 | 0.1746 | 0.2832 | -0.3747 | 0.0617 | -0.2430 |
| Bio17 | 0.1880 | 0.3310 | -0.3791 | -0.0465 | 0.2752 | 0.2887 |
