# Supplementary figures and images for: Potential distribution of Aedes vittatus as an invasive species in North America
Source: PLoS One. 2025 Dec 5;20(12):e0335534. doi: 10.1371/journal.pone.0335534 (PMC12680183; doi:10.1371/journal.pone.0335534)

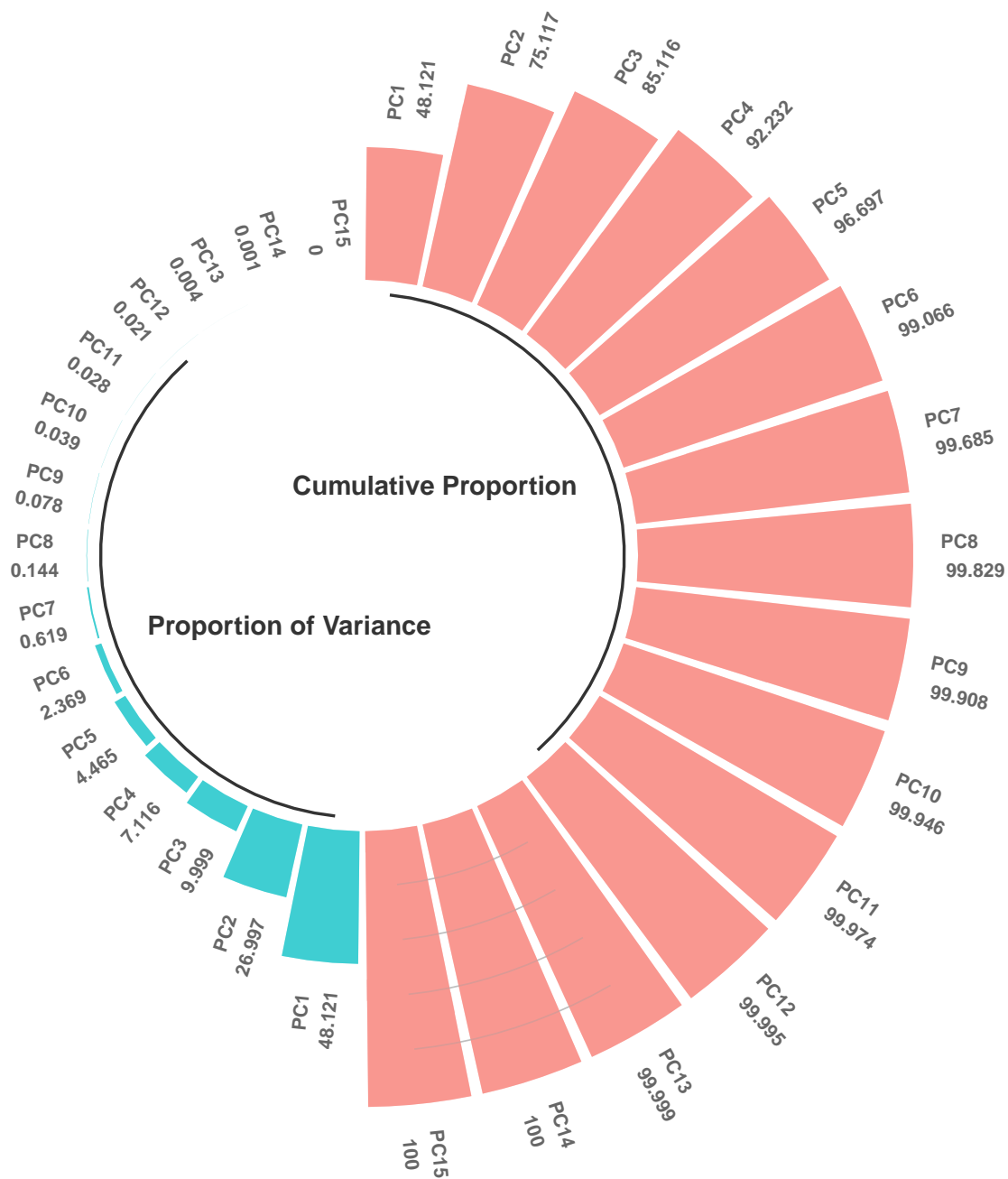

Supplement: S1 Fig — (PDF) [file pone.0335534.s007.pdf]

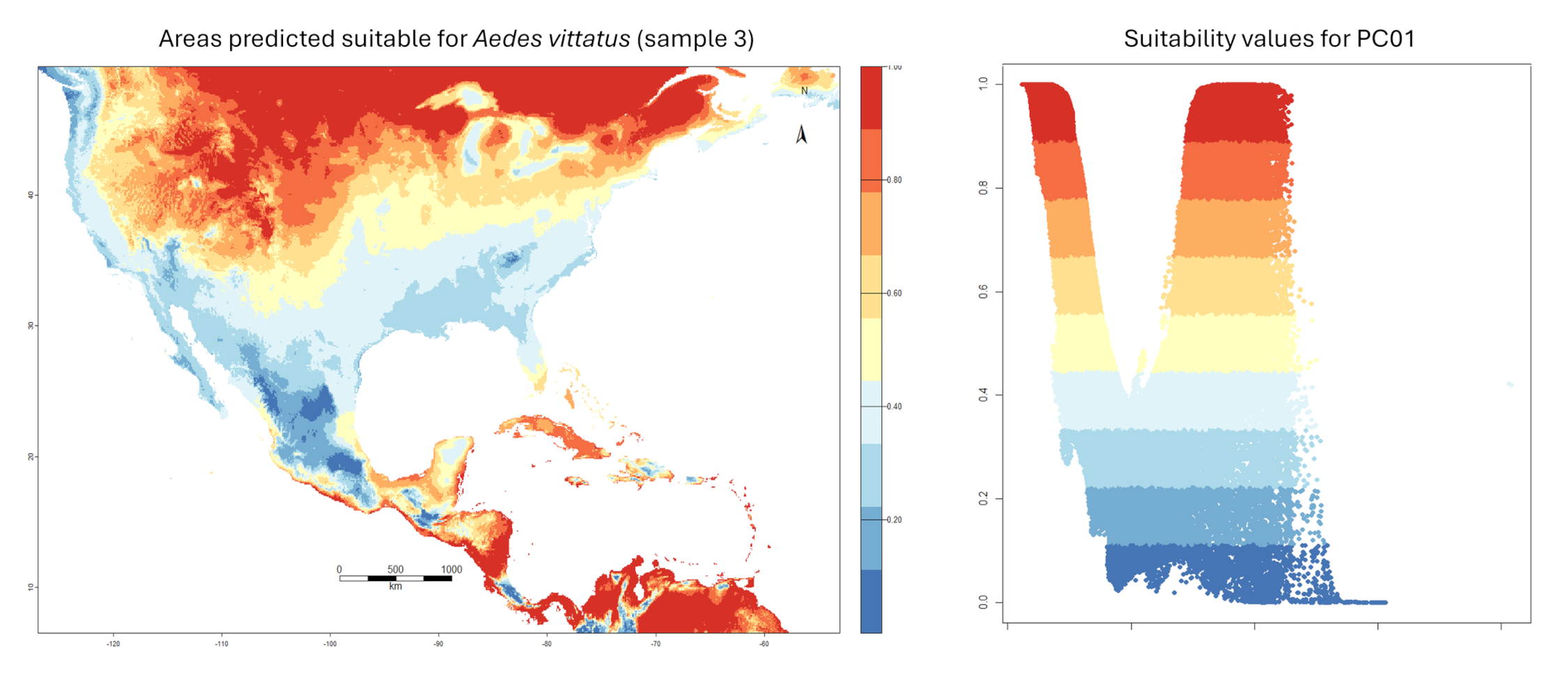

Supplement: S2 Fig — (TIFF) [file pone.0335534.s002.tif]

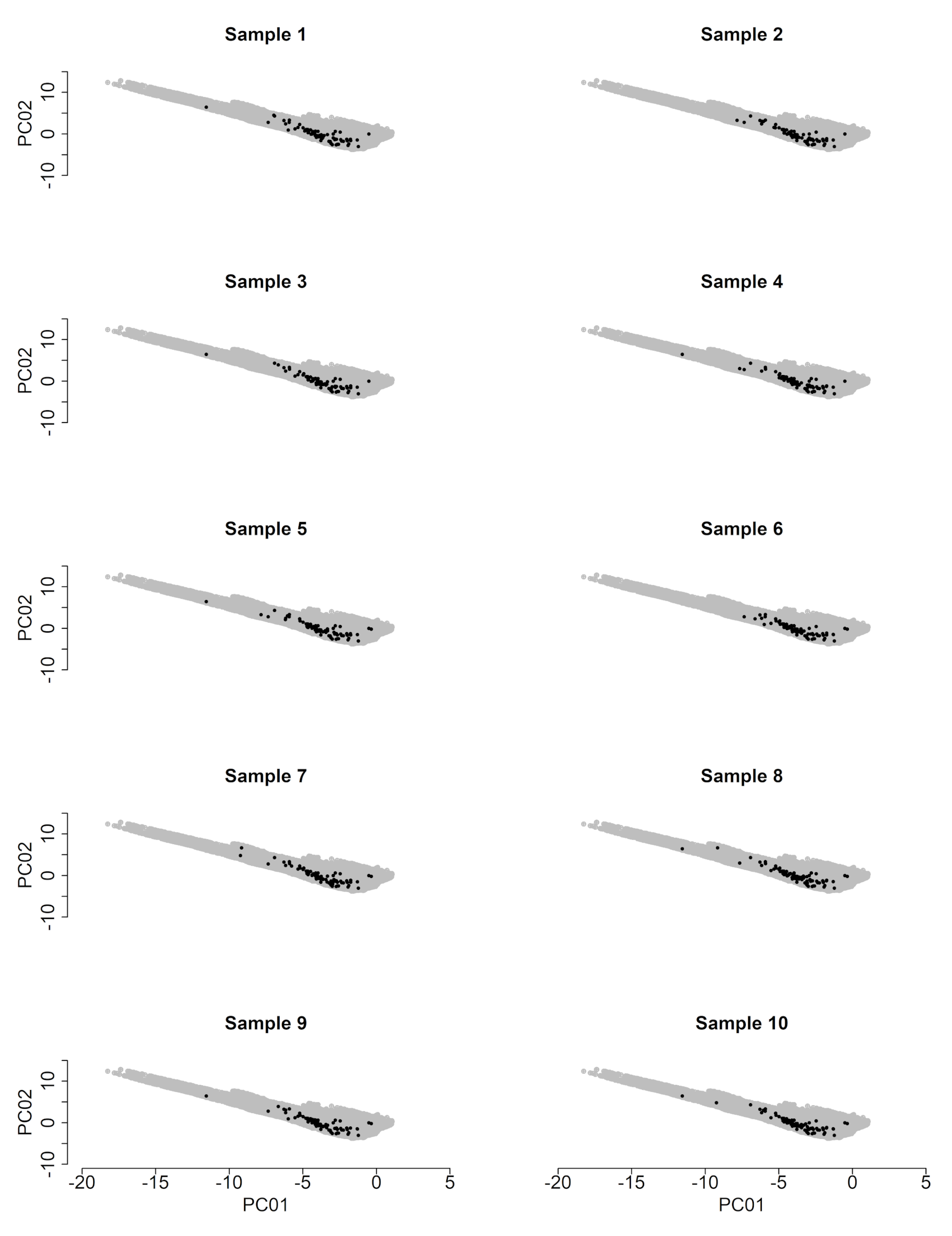

Supplement: S3 Fig — Each plot is one of the randomly sampled data subsets. The plots represent the first principal components (PC) of environments assessed. Each of the dots (gray and black) represents an existing combination of principle components, that is, an existing, unique, environment in the calibration area. The black dots represent the environments of location of reported occurrence of Aedes vittatus. (TIF) [file pone.0335534.s003.tif]

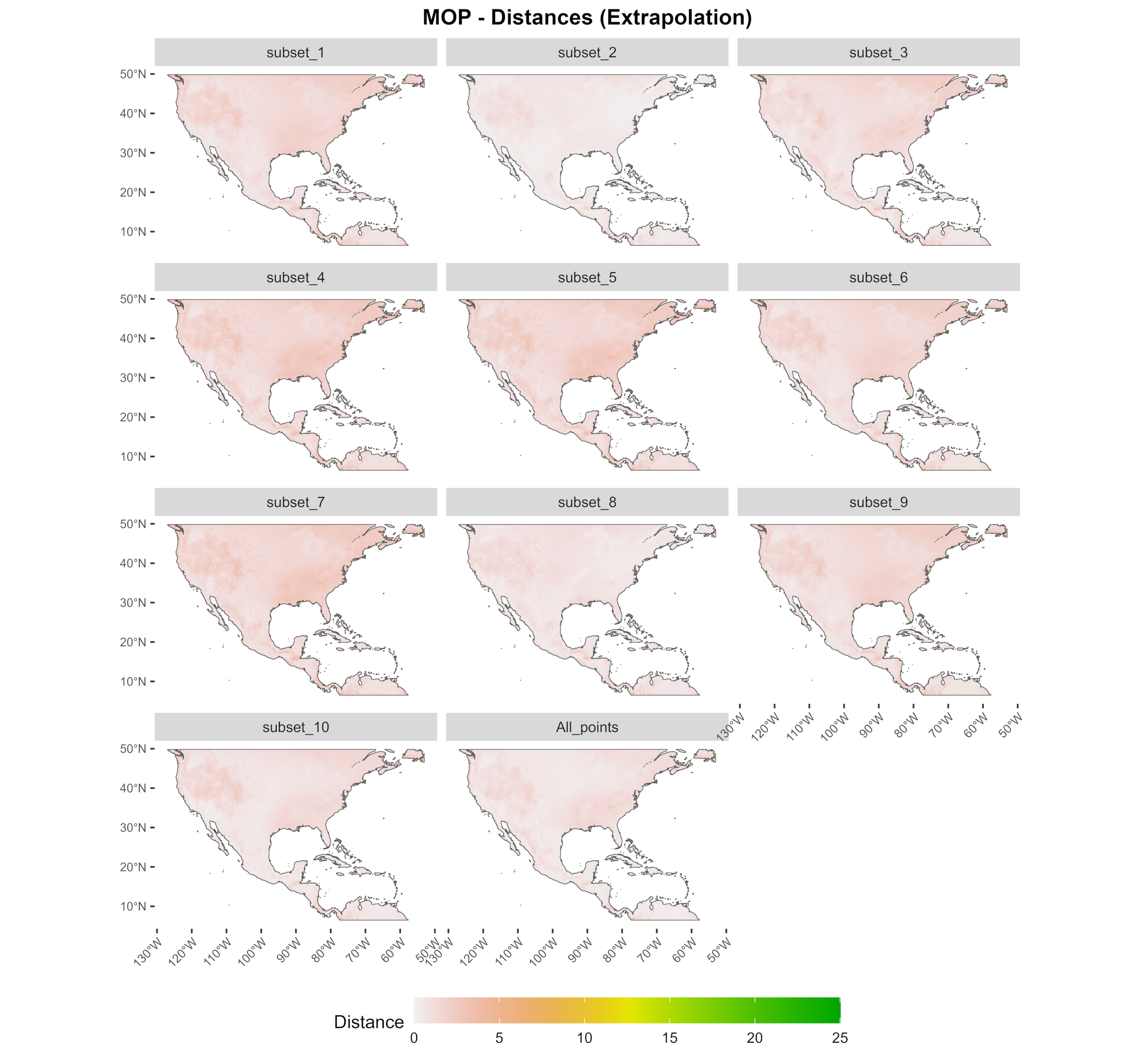

Supplement: S4 Fig — Figure shows estimates of Mobility Oriented-Parity distance which measures the dissimilarity between environments in area of model calibration and transfer. A distance of 0 indicates similar environments while distance of >0 indicate degree of dissimilarity between environments used in model calibration and those in areas of transfer. Basemap data sourced from global administrative areas (GADM), version 4.1, available at www.gadm.org. (TIF) [file pone.0335534.s004.tif]

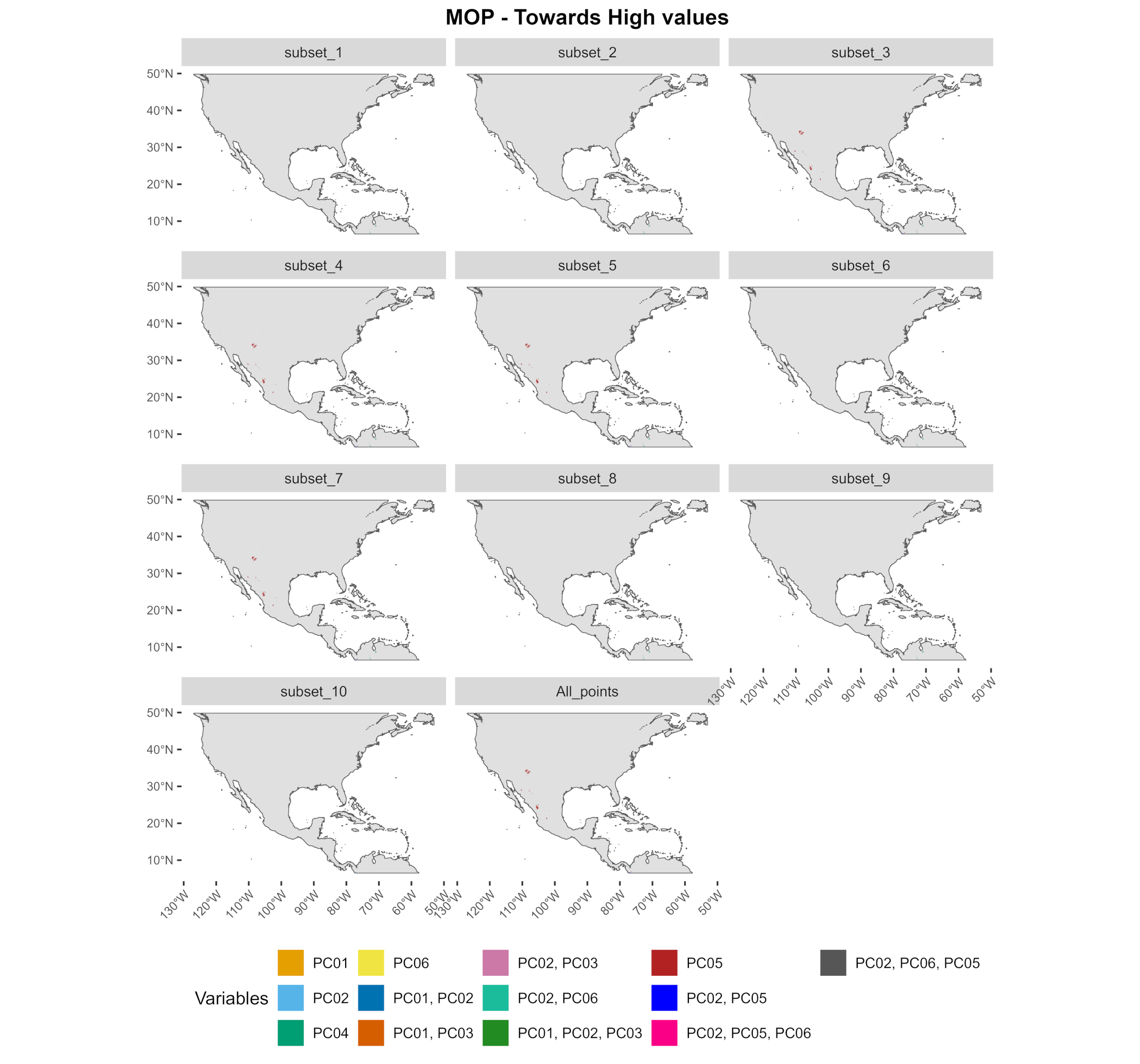

Supplement: S5 Fig — Figure displays dissimilarity estimates of specific environments towards the higher value. Dissimilarity estimates are based on Mobility Oriented-Parity distance. Basemap data sourced from global administrative areas (GADM), version 4.1, available at www.gadm.org. (TIF) [file pone.0335534.s005.tif]

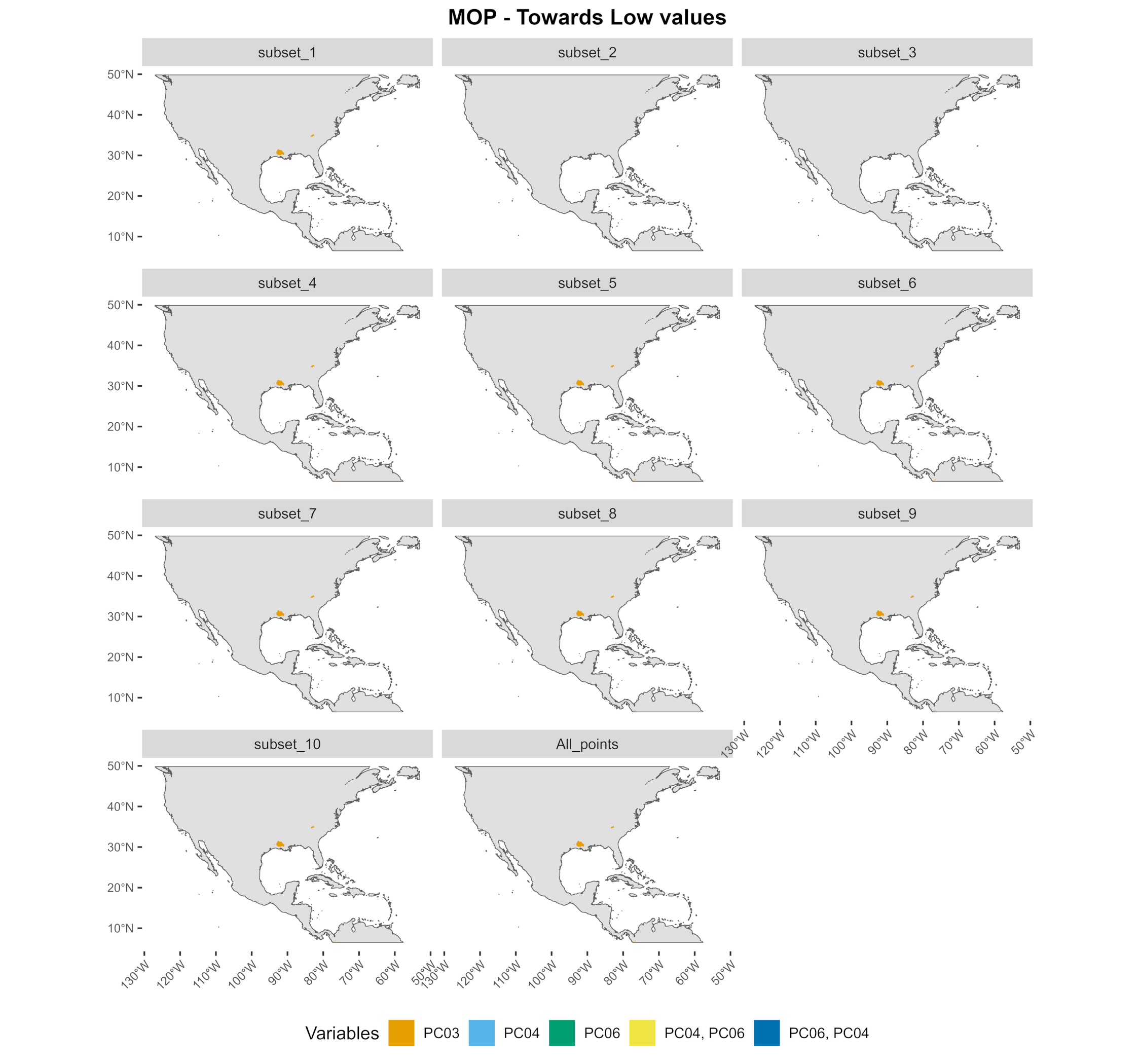

Supplement: S6 Fig — The figure shows dissimilary estimates of specific environments towards the lower value. Dissimilarity estimates are based on Mobility Oriented-Parity distance. Basemap data sourced from global administrative areas (GADM), version 4.1, available at www.gadm.org. (TIF) [file pone.0335534.s006.tif]
